# Some of the Newer Dermatological Remedies and Their Value

**Published:** 1907-11-23

**Authors:** 


### Dermatology.

### THE NEWER DERMATOLOGICAL REMEDIES.

The spirit of conservatism, so characteristic of those who practise the healing art, has caused its influence to be felt in dermatology as much as in other branches of medical science. In one way this is not altogether a disadvantage, for a salutary check is thus provided against the too rapid or wholesale adoption of insufficiently tested therapeutic methods. While it is not evidence of progress to decry the value of every established remedy for skin diseases simply because it does not happen to be the very latest synthetic chemical product, it must yet be recognised that in certain directions real advances in treatment have taken place. The days are long past, it is hoped, when zinc ointment externally and arsenic internally were the sum-total of treatment for every skin case. How different it all is now! The carefully prepared lotion, nicely tempered to suit the degree of inflammatory reaction; the keratolytic plaster or paste for loosening and getting rid of proliferating horny cells; the determined yet discriminating caustic, with its special affinity for attacking disease-foci while leaving healthy skin soundly alone; the beneficent healing and cicatrising effect of the Finsen light and the *x*-rays in many cutaneous disorders, and the employment of the latter for ringworm of the scalp; the improved methods for the administration of mercury in syphilis; and, more recently still, the introduction, by cataphoresis, of certain chemical "ions" for rodent ulcer and a few other affections. These, and many more besides, are among the ways and means which the modern dermatologist has at his disposal for the adequate treatment of skin diseases.

Since the introduction of ichthyol into therapeutics by Unna this drug has continued to rise in popularity and usefulness for many skin affections. There is no denying the fact that the skin tolerates sulphur when organically combined better than when employed in its elemental form. It is not at all uncommon to meet with bad cases of general dermatitis due to the too vigorous rubbing in of the ordinary sulphur ointment, whereas the ichthyol-sulphonate of ammonium does not so easily produce inflammatory reaction. Ichthyol, in addition to being strongly anti-parasitic, has the valuable properties of being able to reduce inflammation, and therefore to relieve pain and tension in the affected parts. For this purpose it may be painted upon the diseased area, *e.g.* a patch of subacute eczema, with a brush, using one drachm to the ounce of water. A favourite application is a 5 per cent. salve, with Lassar's paste, or equal parts of the ung. zinci B.P., and lanoline, as the basis. In chronic eczema it may be used stronger, and in combination with a little salicylic acid. In rosacea the good effect of the familiar compound sulphur ointment, so frequently prescribed, appears to be enhanced by the addition to it of a small quantity of ichthyol.

The tonic effect of the drug upon the vaso-motor system renders it of further use in those conditions accompanied by morbid flushing, and urticarial or erythematous phenomena. It is convenient to administer it in pill form, beginning with a small dose, two minims, thrice daily, after meals, increasing up to five minims. Common acne, many of the erythemata, chronic urticaria, including the giant form, and especially rosacea, are nearly always benefited by ichthyol internally. The sodium salt of the sulphonic acid of a synthetically-prepared sulpho-oil, known as thigenol, resembles ichthyol in many ways, but it has the advantage of being free from the peculiar odour and taste of the latter. It is useful in acute or chronic eczema, and scabies, combining well with sulphur ointment, lanoline, or vaseline. The external application of ichthyol is also recommended for the treatment of chilblains, an ointment containing half-a-drachm to the ounce being rubbed in at night. If ulceration is extensive the dressing is kept on continuously.

The idea of employing brewer's yeast as a remedy for boils is not new. It had long been popularly used in this connection, but Drs. Lassar and Brocq were the first to introduce it seriously and systematically in furunculosis. Even now one hears of stout being recommended for these conditions, not for any stimulant effect, but solely on account of the traces of yeast which it may contain. The specifically active constituent of yeast, a fatty substance, has been isolated by Roos and Hinsberg, and extensive trials have been made with it. Under the name of ceredin (or cerolin) this substance has now been used by many observers, both in this country and abroad, and some really good results have been obtained with it in bad cases of acne pustulosa and furunculosis. It is interesting to note that Dr. Walter Malden, as the outcome of a research undertaken under the auspices of the British Medical Association, found that the effect of subcutaneous injections of yeast upon animals infected with the streptococcus pyogenes or the staphylococcus albus was, in some cases, to cause a recovery of the animals, and in others to prolong their lives. It is best given in pills, containing one-and-a-half grains, commencing with two a day, after meals, and gradually increasing the number according to the effect produced. One point about ceredin is worth remembering; namely, that under its use the tendency of boils to relapse seems to be much diminished.

The beneficial effect of nascent oxygen upon suppurating wounds and indolent ulcerations has long been known. The difficulty and expense of applying it are largely overcome by the use of the peroxides of the metals or of hydrogen. Common ulcers of the leg do very well under applications of zinc peroxide which, in contact with granulation-tissue, slowly evolves oxygen and is itself reduced to the simple oxide. A stimulating, followed by a sedative, effect can thus be produced at will, the salt being employed in ointment form. Hydrogen peroxide, though more active, is sometimes rather too irritating. The sodden, macerated condition about the feet associated with hyperidrosis can be pleasantly relieved by zinc peroxide.

